# Supplementary material for: Randomised controlled trial of population screening for atrial fibrillation in people aged 70 years and over to reduce stroke: protocol for the SAFER trial
Source: BMJ Open. 2024 Apr 25;14(4):e082047. doi: 10.1136/bmjopen-2023-082047 (PMC11057258; doi:10.1136/bmjopen-2023-082047)
Supplement: Author's manuscript [file bmjopen-2023-082047.draft_revisions.pdf]

BMJ Open is committed to open peer review. As part of this commitment we make the peer review history of every article we publish publicly available.

When an article is published we post the peer reviewers' comments and the authors' responses online. We also post the versions of the paper that were used during peer review. These are the versions that the peer review comments apply to.

The versions of the paper that follow are the versions that were submitted during the peer review process. They are not the versions of record or the final published versions. They should not be cited or distributed as the published version of this manuscript.

BMJ Open is an open access journal and the full, final, typeset and author-corrected version of record of the manuscript is available on our site with no access controls, subscription charges or pay-per-view fees (http://bmjopen.bmj.com).

If you have any questions on BMJ Open's open peer review process please email info.bmjopen@bmj.com

BMJ Open

Randomised controlled trial of screening for atrial fibrillation in people aged 70 years and over to reduce stroke: protocol for the SAFER trial.

Journal:	*BMJ Open*
Manuscript ID	bmjopen-2023-082047
Article Type:	Protocol
Date Submitted by the Author:	13-Nov-2023

Complete List of Authors:	Mant, Jonathan; Strangeways Research Laboratory, Primary Care Unit, University of Cambridge<br>Modi, Rakesh; Strangeways Research Laboratory, Primary Care Unit, University of Cambridge<br>Dymond, Andrew; Strangeways Research Laboratory, Primary Care Unit, University of Cambridge<br>Armstrong, Natalie; University of Leicester Department of Population Health Sciences, Department of Population Health Sciences<br>Burt, Jenni; THIS labs<br>Calvert, Peter; Liverpool Heart and Chest Hospital NHS Foundation Trust<br>Cowie, Martin; King's College London School of Cardiovascular and Metabolic Medicine & Sciences<br>Ding, Wern; Liverpool Heart and Chest Hospital NHS Foundation Trust<br>Edwards, Duncan; Strangeways Research Laboratory, Primary Care Unit, University of Cambridge<br>Freedman, Ben; The University of Sydney Charles Perkins Centre<br>Griffin, Simon; University of Cambridge Primary Care Unit, Institute of Public Health; MRC Epidemiology Unit<br>Hoare, Sarah ; University of Cambridge Primary Care Unit, Department of Public Health and Primary Care<br>Hobbs, Richard; University of Oxford Nuffield Department of Primary Care Health Sciences<br>Johnson, Rachel; University of Bristol<br>Kaptoge, Stephen; Cambridge Biomedical Campus<br>Lip, Gregory; Liverpool Heart and Chest Hospital NHS Foundation Trust; Aalborg University Department of Clinical Medicine, Danish Centre for Health Services Research<br>Lobban, Trudie; AF Association, Arrhythmia Alliance and AF association<br>Lown, Mark; University of Southampton School of Primary Care Population Sciences and Medical Education, Primary Care and Population Sciences<br>Lund, Jenny; Strangeways Research Laboratory, Primary Care Unit, Department of Public Health & Primary Care<br>McManus, Richard; University of Oxford Nuffield Department of Primary Care Health Sciences<br>Mills, Mark; Liverpool Heart and Chest Hospital NHS Foundation Trust<br>Morris, Stephen; University of Cambridge Primary Care Unit, Department of Public Health and Primary Care<br>Powell, Alison; THIS Institute, University of Cambridge

	Proietti, Riccardo; Liverpool Heart and Chest Hospital NHS Foundation Trust<br>Sutton, Stephen; University of Cambridge Primary Care Unit, Department of Public Health and Primary Care<br>Sweeting, Mike; Cambridge Biomedical Campus<br>Thom, Howard; University of Bristol<br>Williams, Kate; Strangeways Research Laboratory, Primary Care Unit, University of Cambridge<br>SAFER Authorship Group, The; University of Cambridge Primary Care Unit
Keywords:	Primary Care < Primary Health Care, Mass Screening, Stroke < NEUROLOGY, Randomized Controlled Trial, CARDIOLOGY

Randomised controlled trial of screening for atrial fibrillation in people aged 70 years and over to reduce stroke: protocol for the SAFER trial.

Corresponding author

Dr Rakesh N Modi

Primary Care Unit, Department of Public Health and Primary Care, University of Cambridge, Strangeways Research Laboratory, 2 Worts' Causeway, Cambridge, UK, CB1 8RN

rnm30@medschl.cam.ac.uk

Authors

1. Professor Jonathan Mant, jm677@medschl.cam.ac.uk[1]
2. Dr Rakesh N Modi, rnm30@medschl.cam.ac.uk[1]
3. Andrew Dymond, ad486@medschl.cam.ac.uk[1]
4. Professor Natalie Armstrong, natalie.armstrong@le.ac.uk[2]
5. Dr Jenni Burt, jenni.burt@thislabs.org[3]
6. Dr Peter Calvert, Peter.Calvert@lhch.nhs.uk[4]
7. Professor Martin Cowie, martin.cowie@astrazeneca.com[5]
8. Dr Wern Ding, dwyew@hotmail.com[4]
9. Dr Duncan Edwards, dae31@medschl.cam.ac.uk[1]
10. Professor Ben Freedman, ben.freedman@sydney.edu.au[6]
11. Professor Simon J Griffin, profgp@medschl.cam.ac.uk[1, 7]
12. Dr Sarah Hoare, seh91@medschl.cam.ac.uk[1]
13. Professor F.D. Richard Hobbs, richard.hobbs@phc.ox.ac.uk[8]
14. Dr Rachel Johnson, rachel.johnson@bristol.ac.uk[9]
15. Dr Stephen Kaptoge, skk22@medschl.cam.ac.uk[10]
16. Professor Gregory Y.H. Lip, gregory.lip@liverpool.ac.uk[4]

For peer review only - http://bmjopen.bmj.com/site/about/guidelines.xhtml

17. Ms Trudie Lobban, MBE trudie@heartrhythmalliance.org[11]
18. Dr Mark Lown, M.Lown@soton.ac.uk[12]
19. Dr Jenny Lund, jl897@medschl.cam.ac.uk[1]
20. Professor Richard J McManus, richard.mcmanus@phc.ox.ac.uk[8]
21. Dr Mark T. Mills, Mark.Mills@LHCH.nhs.uk[4]
22. Professor Stephen Morris, sm2428@medschl.cam.ac.uk[1]
23. Dr Alison Powell, alison.powell@thisinstitute.cam.ac.uk[13]
24. Dr Riccardo Proietti, Riccardo.Proietti@liverpool.ac.uk[4]
25. Professor Stephen Sutton, srs34@medschl.cam.ac.uk[1]
26. Dr Mike Sweeting, michael.sweeting@astrazeneca.com[14]
27. Dr Howard Thom, howard.thom@bristol.ac.uk[9]
28. Dr Kate Williams, kmw36@medschl.cam.ac.uk[1]
29. On behalf of the SAFER author group

1. Primary Care Unit, Department of Public Health and Primary Care, University of Cambridge, Strangeways Research Laboratory, 2 Worts' Causeway, Cambridge, UK, CB1 8RN
2. Department of Population Health Sciences, University of Leicester, George Davies Centre, University Road, Leicester, UK, LE1 7RH
3. THIS Labs, 40B Trumpington Mews, High Street, Trumpington, Cambridge, CB2 9LS
4. Liverpool Centre for Cardiovascular Science, University of Liverpool, Liverpool John Moores University and Liverpool Heart & Chest Hospital, Liverpool, UK; Danish Center for Health Services Research, Department of Clinical Medicine, Aalborg University, Denmark
5. School of Cardiovascular Medicine & Sciences, Faculty of Lifesciences & Medicine, King's College London, London, UK
6. Heart Research Institute, University of Sydney, Room 3114, Level 3 East, D17 - Charles Perkins Centre, NSW, Australia, 2006
7. MRC Epidemiology Unit, School of Clinical Medicine, University of Cambridge, Cambridge Biomedical Campus, Cambridge, UK, CB2 0SL
8. Nuffield Department of Primary Care Health Sciences, University of Oxford, Radcliffe Observatory Quarter, Woodstock Road, Oxford, UK, OX2 6GG
9. Bristol Medical School, University of Bristol, Canynge Hall, 39 Whatley Road, Bristol, UK, BS8 2PS
10. Cardiovascular Epidemiology Unit, University of Cambridge, Victor Phillip Dahdaleh Heart & Lung Research Institute, Papworth Road, Cambridge Biomedical Campus, Cambridge, CB2 0BB

11. Arrhythmia Alliance (A-A) and AF Association, Celixir House, Stratford Business &Technology Park Innovation Way, Stratford upon Avon CV37 7GZ
12. Primary Care and Population Sciences, Faculty of Medicine, University of Southampton, Aldermoor Health Centre, Aldermoor Close, Southampton, UK, SO16 5ST
13. THIS Institute (The Healthcare Improvement Studies Institute), University of Cambridge, Strangeways Research Laboratory, 2 Worts Causeway, Cambridge UK CB1 8RN
14. AstraZeneca, 1 Francis Crick Avenue, Cambridge Biomedical Campus, Cambridge, CB2 0AA

For peer review only

ABSTRACT

Introduction

There is a lack of evidence that the benefits of screening for atrial fibrillation (AF) outweigh the harms. Following the completion of the Screening for Atrial Fibrillation with electrocardiogram (ECG) to Reduce stroke (SAFER) pilot trial, the aim of the main SAFER trial is to establish whether screening for AF reduces incidence of stroke risk.

Methods and analysis

Approximately 82,000 people aged 70 years and over and not on oral anticoagulation are being recruited from general practices in England. Patients on the palliative care register or resident in a nursing home are excluded. Eligible people are identified using electronic patient records from general practices and sent an invitation and consent form to participate by post. Consenting participants are randomised at a ratio of 2:1 (control : intervention) with clustering by household. Those randomised to the intervention arm are sent an information leaflet inviting them to participate in screening, which involves use of a handheld single lead ECG four times a day for three weeks. ECG traces identified by an algorithm as possible AF are reviewed by cardiologists. Participants with AF are seen by a general practitioner for consideration of anticoagulation. The primary outcome is stroke. Major secondary outcomes are: death; major bleeding; and cardiovascular events. Follow up will be via electronic health records for an average of four years. The primary analysis will be by intention-to-treat using time-to-event modelling. Results from this trial will be combined with follow up data from the cluster-randomised pilot trial by fixed effect meta-analysis.

Ethics and dissemination

The London—Central Research Ethics Committee (19/LO/1597) provided ethical approval. Dissemination will include public-friendly summaries, reports and engagement with the UK National Screening Committee.

Trial registration number: ISRCTN72104369.

KEYWORDS

Atrial fibrillation; screening; randomised controlled trial; primary care; stroke prevention

For peer review only

ARTICLE SUMMMARY

Strengths and limitations of this study

- This trial is more than twice the size of previous trials of atrial fibrillation (AF) screening and has adequate power to determine whether screening reduces risk of stroke.
- The power calculation has been refined based upon pilot data and the results of an earlier trial which used the same AF screening device.
- The screening intervention has been demonstrated by our feasibility and pilot studies to be feasible for national roll out if shown to be effective.
- There is a risk of contamination in the control group due to increasing availability of personal devices that enable self-screening of AF.
- Outcome data relies on electronic capture of routine data which risks incomplete ascertainment.

For peer review only

INTRODUCTION

The rationale for the Screening for Atrial Fibrillation with ECG to Reduce stroke (SAFER) trial has been described previously.[1] In brief, there is insufficient evidence that the potential benefits from screening for AF outweigh the potential harms.[2] Recent trials have failed to demonstrate that single time-point screening identifies more AF than usual care.[3-5] This is likely to be due to better AF identification within usual care than was prevalent when the Screening for Atrial Fibrillation in the Elderly (SAFE) trial demonstrated the value of single time point screening in identifying additional cases of AF in the early 2000s.[6] Therefore, interest has focussed on newer technologies that enable continuous or intermittent heart rhythm monitoring, such as hand-held ECGs, patches and implantable loop recorders.[7-9] These approaches do identify more AF than usual care, but have not been shown to reduce incidence of stroke.[7-9] Since these devices predominantly identify paroxysmal AF, it is important to determine whether such screening translates into reduced incidence of stroke, as paroxysmal AF may be associated with a lower risk of stroke than permanent AF.[10]

While the evidence base for stroke risk reduction with anticoagulation in AF is based on trials that included participants with paroxysmal AF, the new technologies diagnose people with lower AF burden than will have been typical of those with (usually symptomatic) paroxysmal AF in these trials.[11] Stroke risk in paroxysmal AF is related to AF burden,[12] so it is conceivable that people with low burden paroxysmal AF may not benefit from anticoagulation. Indeed, this was the tentative conclusion drawn by the LOOP study investigators who diagnosed AF in over 30% of the intervention arm of a screening trial using an implantable loop recorder.[8]

The emergence of consumer-led screening over recent years has provided further impetus to the SAFER trial.[13] Several commercially available devices are directly marketed to consumers for detection of AF.[13] The results of SAFER will also guide clinicians on the appropriate course of action in AF identified through consumer-led screening.[13]

In addition to stroke prevention, there are other benefits to treating AF with anticoagulation, including improved survival and reduced risk of myocardial infarction.[11] Indeed, the STROKESTOP screening trial reported a marginally significant reduction in a revised composite primary end-point of stroke, systemic embolism, bleeding leading to hospitalisation and all cause death.[9] Another potential benefit of screening for AF is to reduce risk of cognitive decline and vascular dementia.[14-17]

For peer review only

In terms of harm, the major concern is risk of bleeding as a result of anticoagulation of people identified as being in AF. There is clear evidence in the trials of treatment of AF with anticoagulation that benefit outweighs harm,[11] but the ratio of benefit to harm of treatment might be different for people with AF identified through screening. For example, in the LOOP trial, the 20% relative risk reduction in stroke was largely offset by the 26% relative increase in risk of major bleeding.[8]

The aim of the SAFER trial is to determine if screening for AF using a hand-held single-lead ECG device intermittently over a period of three weeks is effective and cost-effective at reducing stroke compared to usual care and to quantify other potential benefits and harms of screening. The design of the SAFER pilot trial (now successfully completed) has already been reported.[1] This protocol paper therefore focuses on changes in methods between the pilot and the main trial. The SPIRIT checklist when writing this paper.[18]

METHODS AND ANALYSIS

Design

SAFER is a multi-centre randomised controlled trial. Randomisation is at the individual level with clustering by household (i.e., if there is more than one participant from the same address, they will be allocated to the same arm). This is a change from the original intention to randomise at the level of the general practice.[1] This decision was made during the internal pilot trial, when it became clear that remote delivery of the screening intervention greatly reduced the risk of contamination, so negating the value of cluster randomisation by practice. However, it was recognised that there would be a residual risk of contamination if members of the same household were in different arms of the trial. The first patient was randomised in March 2022. It is currently estimated that randomisation will finish in January 2024 and follow-up will finish in March 2027. The trial design is summarised in Figure 1.

<<Figure 1. SAFER trial schema>>

Participants

Participant eligibility is unchanged from the pilot study, being people aged 70 years or older who are registered with participating general practices.[1] Those who are on the practice palliative care

For peer review only

register or in a nursing or residential home are excluded, as are those already on anticoagulation therapy. People with a prior record of AF but not currently on anticoagulation are eligible.[1] General practices are being recruited from throughout England. It is anticipated that about 195 practices will be involved.

Recruitment

Unlike in the pilot cluster randomised trial, where there was little gain in power from increasing sample size in each cluster, all eligible patients (as opposed to a random sample) are sent an invitation pack by their practice. This includes a consent form to be returned to the study team either by post or online.

Randomisation

Randomisation is performed on-line at the Oxford Primary Care Clinical Trials Unit following return of consent forms, stratified by practice. Random permuted blocks ensure allocations are balanced at a ratio of 2:1 (control : intervention) in batches per practice. If there is more than one participant in the same household, they are randomised as a cluster to the same arm.

Baseline data

This is unchanged from the pilot trial, includes demographics and comorbidities, and is collected from the GP electronic medical records.[1]

Screening Intervention

This is unchanged from the pilot trial.[1] In brief, participants randomised to screening will receive a postal invitation to participate. Those who accept this invitation receive a call from the trial team to arrange delivery of the single-lead ECG device (Zenicor) and instructions (written with online video available) and an offer of subsequent support by telephone on how to use it. They are asked to carry out screening four times a day for three weeks, and take additional traces if symptomatic (e.g. palpitations, dizziness). Each trace runs for 30 seconds. Participants transmit their recordings to a remote database using the mobile capability within the device.

For peer review only

A proprietary algorithm (Cardiolund) analyses the ECG traces,[19] and those that show possible AF are reviewed by a cardiologist or cardiac technician. Review by a second cardiologist is performed if there is uncertainty. AF is diagnosed if the rhythm is present continuously for 30 seconds. The results are returned to the practice, which notifies participants of the results, and actively follows up patients with AF or other significant diagnoses (e.g. ventricular tachycardia, high-degree atrioventricular block). Participating GPs receive on line training on the National Institute for Health and Care Excellence (NICE) AF guidelines.[20]

Follow up

The target follow up duration has been reduced from an average of five years (as per the pilot protocol)[1] to four years per participant. This is to compensate for the delays imposed on the trial by COVID-19, and to lower the risk of control group contamination by AF detection device marketing directly to the public.[13] The revised sample size calculation (see below) takes this reduced length of follow up into account. The programme steering committee will review stroke rate in the whole study population (i.e., not by treatment arm), and may recommend modifying follow up duration if stroke rates differ from what is expected. Follow up will be by electronic health records (including GP records), Hospital Episode Statistics, Office for National Statistics mortality data and national disease registries accessed via NHS Digital and ORCHID.[21]

Outcomes

The primary outcome is stroke. This includes stroke of any severity, but excludes events only labelled as transient ischaemic attack. For the primary endpoint, ischaemic and haemorrhagic stroke events will be combined.

Secondary outcomes include: all-cause death; cardiovascular death; major adverse cardiovascular event (composite of myocardial infarction, stroke and other hospital admissions for cardiovascular disease, including heart failure); myocardial infarction; major bleeding episode (defined as requiring hospital admission); new diagnosis of dementia; new diagnosis of depression. AF detection rates and anticoagulation uptake will be reported (principal outcomes of the internal pilot trial).

Sample size

For peer review only

The sample size calculation has been updated to reflect the changes in trial design, the result of a recent trial of screening for AF using the Zenicor device,[9] the interim results of the internal pilot trial, and initial baseline findings from the main trial. In the STROKESTOP trial, an 8% reduction in risk of stroke was observed.[9] Due to higher uptake of screening in the intervention arm of SAFER, and the greater observed differences in AF detection rates between intervention and control as compared to STROKESTOP, a 12% relative risk reduction in stroke is now anticipated in SAFER. Assuming a household cluster size of 1.21 (from observed cluster size to date), a household intraclass correlation coefficient of 0.2,[22] and a 1% annual risk of stroke in the control arm,[9] this equates to needing 82,000 participants to detect a 12% relative reduction in risk of stroke after four years with 90% power.

Analysis

The intention-to-treat principle will guide data analysis (outcome in all randomised participants will be compared between intervention and control). All randomised participants will be included in the analysis, regardless of participation in screening.

The primary analysis will be conducted separately for the cluster randomised pilot trial and the main trial, with results then combined by fixed effect meta-analysis. Time-to-event modelling (i.e. a Cox proportional hazards model) will be used to obtain an estimate (hazard ratio) of the effect of screening on stroke risk (fatal and non-fatal), censoring other causes of death. Analysis time will be from date of randomisation.

Clustering (by practice for pilot trial participants and by household for main trial participants) will be accounted for using a robust sandwich estimator of the covariance matrix. The estimate of intervention effect will be adjusted for pre-specified baseline co-variates such as age and sex. Secondary outcomes will be analysed in a similar way.

For all analyses, we will test model assumptions. Should these be violated, flexible parametric survival models will be considered to model the change in hazard ratio over time.

Economic analysis

For peer review only

To determine whether screening is cost-effective from the perspective of the NHS, we will adapt an existing economic model.[23] This will incorporate data from the SAFER trial, including outcomes such as mortality and cardiovascular endpoints, to determine incremental cost per QALY gained comparing screening versus no screening over a 4 year time horizon. The model parameters that do not come from the trial will be derived from updated literature reviews. We will extend the model to a life-time horizon, and consider the impact on cost-effectiveness of repeated screening at different time intervals and in different age groups.

Management and oversight

Management and oversight is delivered through the same structure as in the pilot trial.[1] The University of Cambridge and NHS Cambridgeshire & Peterborough Integrated Care Board (ICB) are co-sponsors. The trial management group meets monthly to review operational issues. The programme steering committee (PSC), which has an independent chair and four independent members, provides independent over-sight of the programme and acts as the Trial Steering Committee. An active risk register has been compiled in consultation with the funder and sponsors and will be monitored and updated throughout.

Patient and public involvement (PPI)

The same approach is being used as in the pilot trial.[1] In brief, we have engagement by PPI members as an investigator (Trudie Lobban, chief executive of the Atrial Fibrillation Association, (AFA)), and as contributors independent of the AFA.

ETHICS AND DISSEMINATION

Ethical approval has been provided by the London-Central NHS Research Ethics Committee (19/LO/1597).

Public-friendly trial summary documents will be made available to participants at the end of the trial. Accessible reports will be generated for the UK National Screening Committee, commissioners and other decision makers. The pilot study protocol provides further details.[1]

For peer review only

Requests for pseudonymised data will be directed to the study co-ordinator (Andrew Dymond using SAFER@medschl.cam.ac.uk) and will be considered by the investigators, in accordance with participant consent.

AUTHOR CONTRIBUTIONS

JM is the guarantor and drafted the manuscript with help from RNM. KW and AD are coordinating, planning, gaining ethical approval, conducting, and helping design the study. JM, JB, NA, DE, JL, TL, ML, GL, BF, SG, SS, FRH and RJM undertook design, planning and are overseeing conduct of the trial. TL is a PPI representative who has informed the design, outcomes and dissemination plan. SM and HT designed the economic evaluation and will oversee its conduct. SK designed the statistical analysis and will oversee its conduct. GL, PC and RP conducted and refined the cardiology review process of the intervention. The SAFER author group contributed to planning and design of study, applying for funding, writing of the protocol for the ethical approval and have oversight of the conduct of the trial. All authors reviewed and had the option to edit the final manuscript.

FUNDING STATEMENT

This SAFER trial is funded by the National Institute for Health and Care Research (NIHR) [Programme Grants for Applied Research Programme (Reference Number RP-PG-0217-20007)]. The views expressed are those of the author(s) and not necessarily those of the NIHR or the Department of Health and Social Care. SAFER is a contributor to / partner of AFFECT-EU receiving funding from the European Union's Horizon 2020 research and innovation Programme under grant agreement NO. 847770. RM and JL are supported by the Wellcome Trust as part of the Wellcome Trust PhD Programme for Primary Care Clinicians [grant number 203921/Z/16/Z]. AP is based in The Healthcare Improvement Studies Institute (THIS Institute), University of Cambridge. THIS Institute is supported by the Health Foundation, an independent charity committed to bringing about better health and healthcare for people in the UK. FDRH acknowledges support from NIHR ARC OTV and Oxford BRC (OUT). RJM is an NIHR Senior Investigator and acknowledges support from NIHR ARC OTV. NA is supported by a Health Foundation Improvement Science Fellowship and also by the NIHR Applied Research Collaboration East Midlands (ARC EM). RJ is an NIHR-funded Academic Clinical Lecturer. The University of Cambridge has received salary support in respect of SJG from the NHS in the East of England through the Clinical Academic Reserve. BF received funding from the Medical Research Future Fund International Clinical Trial Collaboration Grant to perform SAFER-AUS as part of SAFER,

For peer review only

and a NSW Health Senior Researcher Cardiovascular Grant for work in AF. GYHL is a National Institute for Health and Care Research (NIHR) Senior Investigator and co-principal investigator of the AFFIRMO project on multimorbidity in AF, which has received funding from the European Union's Horizon 2020 research and innovation programme under grant agreement No 899871. All the funders and sponsors had no involvement in the development of this protocol and will have no involvement in any aspect of the study itself. The views expressed are those of the author(s) and not necessarily those of the NHS, the Wellcome Trust, the NIHR or the UK Department of Health and Social Care.

COMPETING INTEREST STATEMENT

JM has performed consultancy work for BMS/Pfizer and Omron. FDRH reports occasional consultancy for BMS/Pfizer, Bayer and BI over the past 5 years. NA is a member of the UK National Screening Committee. MRC and MS are employed by Astrazeneca PLC. RJM's employer the University of Oxford receives consultancy and licencing payments from Omron and Sensyne for BP telemonitoring interventions. GYHL is a consultant and speaker for BMS/Pfizer, Boehringer Ingelheim, Daiichi-Sankyo, Anthos. No fees are received personally. SJG has received honoraria from Astra Zeneca for lectures at postgraduate educational meetings for primary care teams about type 2 diabetes. BF has received speaker fees, honoraria, and non-financial support from the BMS and Pfizer Alliance; grants to the Institution for investigator-initiated studies from the BMS and Pfizer Alliance; and loan devices for investigator-initiated studies from Alivecor: all were unrelated to the present study but related to screening for AF.

ACKNOWLEDGEMENTS

We would like to acknowledge the support of:

Independent Programme Steering Committee: Professor Christian Mallen, University of Keele (chair); Professor Anthony Rudd, Kings College London (independent member); Professor Ann Marie Swart, University of East Anglia (independent member); Professor Andy Vail, University of Manchester (independent member); Dr Bob Ward (independent lay member)

Patient and public involvement representatives: Margaret Corbett; Jennifer Crockford; Trudie Lobban MBE (Founder & CEO of Atrial Fibrillation Association); Sheilah Rengert; Dr Bob Ward

For peer review only

REFERENCES

1. Williams K, Modi RN, Dymond A, et al. Cluster randomised controlled trial of screening for atrial fibrillation in people aged 70 years and over to reduce stroke: protocol for the pilot study for the SAFER trial. *BMJ Open* 2022;12(9):e065066. doi: 10.1136/bmjopen-2022-065066
2. Curry SJ, Krist AH, Owens DK, et al. Screening for Atrial Fibrillation With Electrocardiography: US Preventive Services Task Force Recommendation Statement. *Jama* 2018;320(5):478-84. doi: 10.1001/jama.2018.10321 [published Online First: 2018/08/09]
3. Kaasenbrood F, Hollander M, de Bruijn SH, et al. Opportunistic screening versus usual care for diagnosing atrial fibrillation in general practice: a cluster randomised controlled trial. *Br J Gen Pract* 2020;70(695):e427-e33. doi: 10.3399/bjgp20X708161 [published Online First: 2020/01/29]
4. Uittenbogaart SB, Verbiest-van Gurp N, Lucassen WAM, et al. Opportunistic screening versus usual care for detection of atrial fibrillation in primary care: cluster randomised controlled trial. *BMJ* 2020;370:m3208. doi: 10.1136/bmj.m3208
5. Lubitz SA, Atlas SJ, Ashburner JM, et al. Screening for Atrial Fibrillation in Older Adults at Primary Care Visits: VITAL-AF Randomized Controlled Trial. *Circulation* 2022;145(13):946-54. doi: doi:10.1161/CIRCULATIONAHA.121.057014
6. Fitzmaurice DA, Hobbs FD, Jowett S, et al. Screening versus routine practice in detection of atrial fibrillation in patients aged 65 or over: cluster randomised controlled trial. *Bmj* 2007;335(7616):383. doi: 10.1136/bmj.39280.660567.55 [published Online First: 2007/08/04]
7. Gladstone DJ, Wachter R, Schmalstieg-Bahr K, et al. Screening for Atrial Fibrillation in the Older Population: A Randomized Clinical Trial. *JAMA Cardiology* 2021 doi: 10.1001/jamacardio.2021.0038
8. Svendsen JH, Diederichsen SZ, Højberg S, et al. Implantable loop recorder detection of atrial fibrillation to prevent stroke (The LOOP Study): a randomised controlled trial. *The Lancet* 2021 doi: https://doi.org/10.1016/S0140-6736(21)01698-6
9. Svennberg E, Friberg L, Frykman V, et al. Clinical outcomes in systematic screening for atrial fibrillation (STROKESTOP): a multicentre, parallel group, unmasked, randomised controlled trial. *The Lancet* 2021 doi: https://doi.org/10.1016/S0140-6736(21)01637-8
10. Ganesan AN, Chew DP, Hartshorne T, et al. The impact of atrial fibrillation type on the risk of thromboembolism, mortality, and bleeding: a systematic review and meta-analysis. *Eur Heart J* 2016;37(20):1591-602. doi: 10.1093/eurheartj/ehw007 [published Online First: 2016/02/19]
11. Aguilar MI, Hart R. Oral anticoagulants for preventing stroke in patients with non-valvular atrial fibrillation and no previous history of stroke or transient ischemic attacks. *Cochrane Database of Systematic Reviews* 2005(3) doi: 10.1002/14651858.CD001927.pub2
12. Go AS, Reynolds K, Yang J, et al. Association of Burden of Atrial Fibrillation With Risk of Ischemic Stroke in Adults With Paroxysmal Atrial Fibrillation: The KP-RHYTHM Study. *JAMA Cardiology* 2018;3(7):601-08. doi: 10.1001/jamacardio.2018.1176
13. Brandes A, Stavrakis S, Freedman B, et al. Consumer-Led Screening for Atrial Fibrillation: Frontier Review of the AF-SCREEN International Collaboration. *Circulation* 2022;146(19):1461-74. doi: 10.1161/circulationaha.121.058911 [published Online First: 2022/11/08]
14. Singh-Manoux A, Fayosse A, Sabia S, et al. Atrial fibrillation as a risk factor for cognitive decline and dementia. *European Heart Journal* 2017;38(34):2612-18.
15. Bezabhe WM, Bereznicki LR, Radford J, et al. Oral Anticoagulant Treatment and the Risk of Dementia in Patients With Atrial Fibrillation: A Population-Based Cohort Study. *Journal of the American Heart Association* 2022;11(7):e023098. doi: doi:10.1161/JAHA.121.023098

For peer review only

16. Friberg L, Andersson T, Rosenqvist M. Less dementia and stroke in low-risk patients with atrial fibrillation taking oral anticoagulation. *European Heart Journal* 2019;40(28):2327-35. doi: 10.1093/eurheartj/ehz304
17. Mavaddat N, Roalfe A, Fletcher K, et al. Warfarin versus aspirin for prevention of cognitive decline in atrial fibrillation: randomized controlled trial (Birmingham Atrial Fibrillation Treatment of the Aged Study). *Stroke* 2014;45(5):1381-86.
18. Chan AW, Tetzlaff JM, Gøtzsche PC, et al. SPIRIT 2013 explanation and elaboration: guidance for protocols of clinical trials. *Bmj* 2013;346:e7586. doi: 10.1136/bmj.e7586 [published Online First: 2013/01/11]
19. Cardiolund. Cardiac Analysis 2023 [Available from: https://www.cardiolund.com/ accessed 31/07/2023.
20. National Institute for Health and Care Excellence. Atrial fibrillation: diagnosis and management (ng196): National Institute of Health and Care Excellence, 2021.
21. Nuffield Department of Primary Care Health Sciences. ORCHID: University of Oxford; 2023 [Available from: https://orchid.phc.ox.ac.uk/ accessed 13/11/2023.
22. Ukoumunne OC, Gulliford MC, Chinn S, et al. Methods for evaluating area-wide and organisation-based interventions in health and health care: a systematic review. *Health technology assessment (Winchester, England)* 1999;3(5):iii-92. [published Online First: 2000/09/12]
23. Welton NJ, McAleenan A, Thom HH, et al. Screening strategies for atrial fibrillation: a systematic review and cost-effectiveness analysis. *Health Technology Assessment* 2017;21(29)

For peer review only

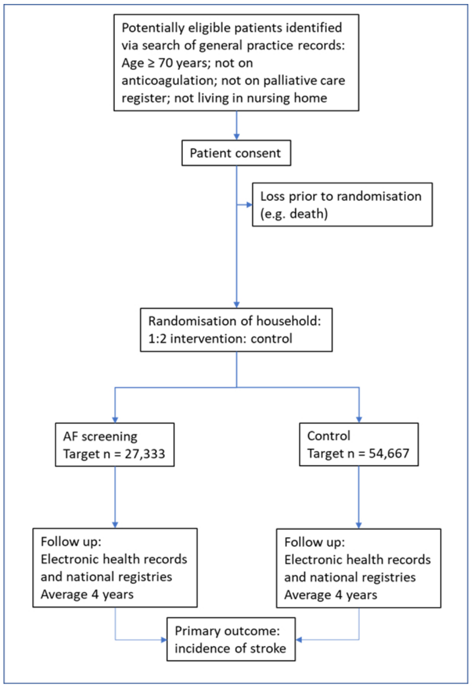

Figure 1. SAFER trial schema

225x180mm (144 x 144 DPI)

Reporting checklist for protocol of a clinical trial.

Based on the SPIRIT guidelines.

Administrative information	Reporting Item	Page Number
Title	Descriptive title identifying the study design, population, interventions, and, if applicable, trial acronym	1
Trial registration	Trial identifier and registry name. If not yet registered, name of intended registry	4
Trial registration: data set	All items from the World Health Organization Trial Registration Data Set	4
Protocol version	Date and version identifier	n/a
Funding	Sources and types of financial, material, and other support	12-13
Roles and responsibilities: contributorship	Names, affiliations, and roles of protocol contributors	1-3, 12
Roles and responsibilities: sponsor contact information	Name and contact information for the trial sponsor	11

For peer review only

Roles and responsibilities: sponsor and funder	Role of study sponsor and funders, if any, in study design; collection, management, analysis, and interpretation of data; writing of the report; and the decision to submit the report for publication, including whether they will have ultimate authority over any of these activities	11-12
Roles and responsibilities: committees	Composition, roles, and responsibilities of the coordinating centre, steering committee, endpoint adjudication committee, data management team, and other individuals or groups overseeing the trial, if applicable (see Item 21a for data monitoring committee)	11, 13
**Introduction**		
Background and rationale	Description of research question and justification for undertaking the trial, including summary of relevant studies (published and unpublished) examining benefits and harms for each intervention	6-7
Background and rationale: choice of comparators	Explanation for choice of comparators	6-9
Objectives	Specific objectives or hypotheses	7
Trial design	Description of trial design including type of trial (eg, parallel group, crossover, factorial, single group), allocation ratio, and framework (eg, superiority, equivalence, non-inferiority, exploratory)	7

For peer review only

**Methods: Participants, interventions, and outcomes**		
Study setting	Description of study settings (eg, community clinic, academic hospital) and list of countries where data will be collected. Reference to where list of study sites can be obtained	7-8, 12
Eligibility criteria	Inclusion and exclusion criteria for participants. If applicable, eligibility criteria for study centres and individuals who will perform the interventions (eg, surgeons, psychotherapists)	7-8
Interventions: description	Interventions for each group with sufficient detail to allow replication, including how and when they will be administered	8-9
Interventions: modifications	Criteria for discontinuing or modifying allocated interventions for a given trial participant (eg, drug dose change in response to harms, participant request, or improving / worsening disease)	8-9
Interventions: adherance	Strategies to improve adherence to intervention protocols, and any procedures for monitoring adherence (eg, drug tablet return; laboratory tests)	9
Interventions: concomitant care	Relevant concomitant care and interventions that are permitted or prohibited during the trial	8-9

For peer review only

Outcomes	Primary, secondary, and other outcomes, including the specific measurement variable (eg, systolic blood pressure), analysis metric (eg, change from baseline, final value, time to event), method of aggregation (eg, median, proportion), and time point for each outcome. Explanation of the clinical relevance of chosen efficacy and harm outcomes is strongly recommended	9
Participant timeline	Time schedule of enrolment, interventions (including any run-ins and washouts), assessments, and visits for participants. A schematic diagram is highly recommended (see Figure)	8-9
Sample size	Estimated number of participants needed to achieve study objectives and how it was determined, including clinical and statistical assumptions supporting any sample size calculations	9-10
Recruitment	Strategies for achieving adequate participant enrolment to reach target sample size	7-8
**Methods: Assignment of interventions (for controlled trials)**		
Allocation: sequence generation	Method of generating the allocation sequence (eg, computer-generated random numbers), and list of any factors for stratification. To reduce predictability of a random sequence, details of any planned restriction (eg,	8

For peer review only

	blocking) should be provided in a separate document that is unavailable to those who enrol participants or assign interventions	
Allocation concealment mechanism	Mechanism of implementing the allocation sequence (eg, central telephone; sequentially numbered, opaque, sealed envelopes), describing any steps to conceal the sequence until interventions are assigned	n/a
Allocation: implementation	Who will generate the allocation sequence, who will enrol participants, and who will assign participants to interventions	8
Blinding (masking)	Who will be blinded after assignment to interventions (eg, trial participants, care providers, outcome assessors, data analysts), and how	n/a
Blinding (masking): emergency unblinding	If blinded, circumstances under which unblinding is permissible, and procedure for revealing a participant's allocated intervention during the trial	n/a
**Methods: Data collection, management, and analysis**		
Data collection plan	Plans for assessment and collection of outcome, baseline, and other trial data, including any related processes to promote data quality (eg, duplicate measurements, training of assessors) and a description of study instruments (eg, questionnaires, laboratory tests) along	8-9

For peer review only

	with their reliability and validity, if known. Reference to where data collection forms can be found, if not in the protocol	
Data collection plan: retention	Plans to promote participant retention and complete follow-up, including list of any outcome data to be collected for participants who discontinue or deviate from intervention protocols	9
Data management	Plans for data entry, coding, security, and storage, including any related processes to promote data quality (eg, double data entry; range checks for data values). Reference to where details of data management procedures can be found, if not in the protocol	8, 11
Statistics: outcomes	Statistical methods for analysing primary and secondary outcomes. Reference to where other details of the statistical analysis plan can be found, if not in the protocol	10-11
Statistics: additional analyses	Methods for any additional analyses (eg, subgroup and adjusted analyses)	10-11
Statistics: analysis population and missing data	Definition of analysis population relating to protocol non-adherence (eg, as randomised analysis), and any statistical methods to handle missing data (eg, multiple imputation)	10
**Methods: Monitoring**		
Data monitoring: formal	Composition of data monitoring committee (DMC);	11, 13

For peer review only

committee	summary of its role and reporting structure; statement of whether it is independent from the sponsor and competing interests; and reference to where further details about its charter can be found, if not in the protocol. Alternatively, an explanation of why a DMC is not needed	
Data monitoring: interim analysis	Description of any interim analyses and stopping guidelines, including who will have access to these interim results and make the final decision to terminate the trial	11, 13
Harms	Plans for collecting, assessing, reporting, and managing solicited and spontaneously reported adverse events and other unintended effects of trial interventions or trial conduct	11
Auditing	Frequency and procedures for auditing trial conduct, if any, and whether the process will be independent from investigators and the sponsor	11
**Ethics and dissemination**		
Research ethics approval	Plans for seeking research ethics committee / institutional review board (REC / IRB) approval	11-12
Protocol amendments	Plans for communicating important protocol modifications (eg, changes to eligibility criteria, outcomes, analyses) to relevant parties (eg, investigators, REC / IRBs, trial participants, trial registries, journals, regulators)	11
Consent or assent	Who will obtain informed consent or assent from potential	8

For peer review only

	trial participants or authorised surrogates, and how (see Item 32)	
Consent or assent: ancillary studies	Additional consent provisions for collection and use of participant data and biological specimens in ancillary studies, if applicable	n/a
Confidentiality	How personal information about potential and enrolled participants will be collected, shared, and maintained in order to protect confidentiality before, during, and after the trial	11-12
Declaration of interests	Financial and other competing interests for principal investigators for the overall trial and each study site	12-13
Data access	Statement of who will have access to the final trial dataset, and disclosure of contractual agreements that limit such access for investigators	12
Ancillary and post trial care	Provisions, if any, for ancillary and post-trial care, and for compensation to those who suffer harm from trial participation	n/a
Dissemination policy: trial results	Plans for investigators and sponsor to communicate trial results to participants, healthcare professionals, the public, and other relevant groups (eg, via publication, reporting in results databases, or other data sharing arrangements), including any publication restrictions	11
Dissemination policy:	Authorship eligibility guidelines and any intended use of	12

For peer review only

authorship	professional writers	
Dissemination policy: reproducible research	Plans, if any, for granting public access to the full protocol, participant-level dataset, and statistical code	12
**Appendices**		
Informed consent materials	Model consent form and other related documentation given to participants and authorised surrogates	n/a
Biological specimens	Plans for collection, laboratory evaluation, and storage of biological specimens for genetic or molecular analysis in the current trial and for future use in ancillary studies, if applicable	n/a

For peer review only

BMJ Open

Randomised controlled trial of population screening for atrial fibrillation in people aged 70 years and over to reduce stroke: protocol for the SAFER trial.

Journal:	*BMJ Open*
Manuscript ID	bmjopen-2023-082047.R1
Article Type:	Protocol
Date Submitted by the Author:	25-Jan-2024
Complete List of Authors:	Mant, Jonathan; Strangeways Research Laboratory, Primary Care Unit, University of Cambridge<br>Modi, Rakesh; Strangeways Research Laboratory, Primary Care Unit, University of Cambridge<br>Dymond, Andrew; Strangeways Research Laboratory, Primary Care Unit, University of Cambridge<br>Armstrong, Natalie; University of Leicester Department of Population Health Sciences, Department of Population Health Sciences<br>Burt, Jenni; THIS labs<br>Calvert, Peter; Liverpool Heart and Chest Hospital NHS Foundation Trust<br>Cowie, Martin; King's College London School of Cardiovascular and Metabolic Medicine & Sciences<br>Ding, Wern; Liverpool Heart and Chest Hospital NHS Foundation Trust<br>Edwards, Duncan; Strangeways Research Laboratory, Primary Care Unit, University of Cambridge<br>Freedman, Ben; The University of Sydney Charles Perkins Centre<br>Griffin, Simon; University of Cambridge Primary Care Unit, Institute of Public Health; MRC Epidemiology Unit<br>Hoare, Sarah ; University of Cambridge Primary Care Unit, Department of Public Health and Primary Care<br>Hobbs, Richard; University of Oxford Nuffield Department of Primary Care Health Sciences<br>Johnson, Rachel; University of Bristol<br>Kaptoge, Stephen; Cambridge Biomedical Campus<br>Lip, Gregory; Liverpool Heart and Chest Hospital NHS Foundation Trust; Aalborg University Department of Clinical Medicine, Danish Centre for Health Services Research<br>Lobban, Trudie; AF Association, Arrhythmia Alliance and AF association<br>Lown, Mark; University of Southampton School of Primary Care Population Sciences and Medical Education, Primary Care and Population Sciences<br>Lund, Jenny; Strangeways Research Laboratory, Primary Care Unit, Department of Public Health & Primary Care<br>McManus, Richard; University of Oxford Nuffield Department of Primary Care Health Sciences<br>Mills, Mark; Liverpool Heart and Chest Hospital NHS Foundation Trust<br>Morris, Stephen; University of Cambridge Primary Care Unit, Department of Public Health and Primary Care<br>Powell, Alison; THIS Institute, University of Cambridge

	Proietti, Riccardo; Liverpool Heart and Chest Hospital NHS Foundation Trust<br>Sutton, Stephen; University of Cambridge Primary Care Unit, Department of Public Health and Primary Care<br>Sweeting, Mike; Cambridge Biomedical Campus<br>Thom, Howard; University of Bristol<br>Williams, Kate; Strangeways Research Laboratory, Primary Care Unit, University of Cambridge<br>SAFER Authorship Group, The; University of Cambridge Primary Care Unit
**Primary Subject Heading**:	Cardiovascular medicine
Secondary Subject Heading:	Public health
Keywords:	Primary Care < Primary Health Care, Mass Screening, Stroke < NEUROLOGY, Randomized Controlled Trial, CARDIOLOGY

Randomised controlled trial of population screening for atrial fibrillation in people aged 70 years and over to reduce stroke: protocol for the SAFER trial.

For peer review

Corresponding author

Dr Rakesh N Modi
Primary Care Unit, Department of Public Health and Primary Care, University of Cambridge, Strangeways Research Laboratory, 2 Worts' Causeway, Cambridge, UK, CB1 8RN
rnm30@medschl.cam.ac.uk

Authors

1. Professor Jonathan Mant, jm677@medschl.cam.ac.uk[1]
2. Dr Rakesh N Modi, rnm30@medschl.cam.ac.uk[1]
3. Andrew Dymond, ad486@medschl.cam.ac.uk[1]
4. Professor Natalie Armstrong, natalie.armstrong@le.ac.uk[2]
5. Dr Jenni Burt, jenni.burt@thislabs.org[3]
6. Dr Peter Calvert, Peter.Calvert@lhch.nhs.uk[4]
7. Professor Martin Cowie, martin.cowie@astrazeneca.com[5]
8. Dr Wern Yew Ding, dwyew@hotmail.com[4]
9. Dr Duncan Edwards, dae31@medschl.cam.ac.uk[1]
10. Professor Ben Freedman, ben.freedman@sydney.edu.au[6]
11. Professor Simon J Griffin, profgp@medschl.cam.ac.uk[1, 7]
12. Dr Sarah Hoare, seh91@medschl.cam.ac.uk[1]
13. Professor F.D. Richard Hobbs, richard.hobbs@phc.ox.ac.uk[8]
14. Dr Rachel Johnson, rachel.johnson@bristol.ac.uk[9]
15. Dr Stephen Kaptoge, skk22@medschl.cam.ac.uk[10]
16. Professor Gregory Y.H. Lip, gregory.lip@liverpool.ac.uk[4]

17. Ms Trudie Lobban, MBE trudie@heartrhythmalliance.org[11]
18. Dr Mark Lown, M.Lown@soton.ac.uk[12]
19. Dr Jenny Lund, jl897@medschl.cam.ac.uk[1]
20. Professor Richard J McManus, richard.mcmanus@phc.ox.ac.uk[8]
21. Dr Mark T. Mills, Mark.Mills@LHCH.nhs.uk[4]
22. Professor Stephen Morris, sm2428@medschl.cam.ac.uk[1]
23. Dr Alison Powell, alison.powell@thisinstitute.cam.ac.uk[13]
24. Dr Riccardo Proietti, Riccardo.Proietti@liverpool.ac.uk[4]
25. Professor Stephen Sutton, srs34@medschl.cam.ac.uk[1]
26. Dr Mike Sweeting, michael.sweeting@astrazeneca.com[2]
27. Dr Howard Thom, howard.thom@bristol.ac.uk[9]
28. Dr Kate Williams, kmw36@medschl.cam.ac.uk[1]
29. On behalf of the SAFER author group

1. Primary Care Unit, Department of Public Health and Primary Care, University of Cambridge, Strangeways Research Laboratory, 2 Worts' Causeway, Cambridge, UK, CB1 8RN
2. Department of Population Health Sciences, University of Leicester, George Davies Centre, University Road, Leicester, UK, LE1 7RH
3. THIS Labs, 40B Trumpington Mews, High Street, Trumpington, Cambridge, CB2 9LS
4. Liverpool Centre for Cardiovascular Science, University of Liverpool, Liverpool John Moores University and Liverpool Heart & Chest Hospital, Liverpool, UK; Danish Center for Health Services Research, Department of Clinical Medicine, Aalborg University, Denmark
5. School of Cardiovascular Medicine & Sciences, Faculty of Lifesciences & Medicine, King's College London, London, UK
6. Heart Research Institute, University of Sydney, Room 3114, Level 3 East, D17 - Charles Perkins Centre, NSW, Australia, 2006
7. MRC Epidemiology Unit, School of Clinical Medicine, University of Cambridge, Cambridge Biomedical Campus, Cambridge, UK, CB2 0SL
8. Nuffield Department of Primary Care Health Sciences, University of Oxford, Radcliffe Observatory Quarter, Woodstock Road, Oxford, UK, OX2 6GG
9. Bristol Medical School, University of Bristol, Canynge Hall, 39 Whatley Road, Bristol, UK, BS8 2PS
10. Cardiovascular Epidemiology Unit, University of Cambridge, Victor Phillip Dahdaleh Heart & Lung Research Institute, Papworth Road, Cambridge Biomedical Campus, Cambridge, CB2 0BB

11. Arrhythmia Alliance (A-A) and AF Association, Celixir House, Stratford Business &Technology Park Innovation Way, Stratford upon Avon CV37 7GZ
12. Primary Care and Population Sciences, Faculty of Medicine, University of Southampton, Aldermoor Health Centre, Aldermoor Close, Southampton, UK, SO16 5ST
13. THIS Institute (The Healthcare Improvement Studies Institute), University of Cambridge, Strangeways Research Laboratory, 2 Worts Causeway, Cambridge UK CB1 8RN

For peer review only

ABSTRACT

Introduction

There is a lack of evidence that the benefits of screening for atrial fibrillation (AF) outweigh the harms. Following the completion of the Screening for Atrial Fibrillation with electrocardiogram (ECG) to Reduce stroke (SAFER) pilot trial, the aim of the main SAFER trial is to establish whether populaton screening for AF reduces incidence of stroke risk.

Methods and analysis

Approximately 82,000 people aged 70 years and over and not on oral anticoagulation are being recruited from general practices in England. Patients on the palliative care register or resident in a nursing home are excluded. Eligible people are identified using electronic patient records from general practices and sent an invitation and consent form to participate by post. Consenting participants are randomised at a ratio of 2:1 (control : intervention) with clustering by household. Those randomised to the intervention arm are sent an information leaflet inviting them to participate in screening, which involves use of a handheld single lead ECG four times a day for three weeks. ECG traces identified by an algorithm as possible AF are reviewed by cardiologists. Participants with AF are seen by a general practitioner for consideration of anticoagulation. The primary outcome is stroke. Major secondary outcomes are: death; major bleeding; and cardiovascular events. Follow up will be via electronic health records for an average of four years. The primary analysis will be by intention-to-treat using time-to-event modelling. Results from this trial will be combined with follow up data from the cluster-randomised pilot trial by fixed effect meta-analysis.

Ethics and dissemination

The London—Central NHS Research Ethics Committee (19/LO/1597) provided ethical approval. Dissemination will include public-friendly summaries, reports and engagement with the UK National Screening Committee.

Trial registration number: ISRCTN72104369.

KEYWORDS

Atrial fibrillation; screening; randomised controlled trial; primary care; stroke prevention

For peer review only

ARTICLE SUMMMARY

Strengths and limitations of this study

- This trial is more than twice the size of previous trials of atrial fibrillation (AF) screening and has adequate power to determine whether screening reduces risk of stroke.
- The power calculation has been refined based upon pilot data and the results of an earlier trial which used the same AF screening device.
- The screening intervention has been demonstrated by our feasibility and pilot studies to be feasible for national roll out if shown to be effective.
- There is a risk of contamination in the control group due to increasing availability of personal devices that enable self-screening for AF.
- Outcome data relies on electronic capture of routine data which risks incomplete ascertainment.

For peer review only

INTRODUCTION

The rationale for the Screening for Atrial Fibrillation with ECG to Reduce stroke (SAFER) trial has been described previously.[1] In brief, there is insufficient evidence that the potential benefits from screening for AF outweigh the potential harms.[2] Recent trials have failed to demonstrate that single time-point screening identifies more AF than usual care.[3-5] This is likely to be due to better AF identification within usual care than was prevalent when the Screening for Atrial Fibrillation in the Elderly (SAFE) trial demonstrated the value of single time point screening in identifying additional cases of AF in the early 2000s.[6] Therefore, interest has focussed on newer technologies that enable continuous or intermittent heart rhythm monitoring, such as hand-held ECGs, patches and implantable loop recorders.[7-9] These approaches do identify more AF than usual care, but have not been shown to reduce incidence of stroke.[7-9] Since these devices predominantly identify paroxysmal AF, it is important to determine whether such screening translates into reduced incidence of stroke, as paroxysmal AF may be associated with a lower risk of stroke than permanent AF.[10]

While the evidence base for stroke risk reduction with anticoagulation in AF is based on trials that included participants with paroxysmal AF, the new technologies diagnose people with lower AF burden than will have been typical of those with (usually symptomatic) paroxysmal AF in these trials.[11] Stroke risk in paroxysmal AF is related to AF burden,[12] so it is conceivable that people with low burden paroxysmal AF may not benefit from anticoagulation. Indeed, this was the tentative conclusion drawn by the LOOP study investigators who diagnosed AF in over 30% of the intervention arm of a screening trial using an implantable loop recorder.[8]

The emergence of consumer-led screening over recent years has provided further impetus to the SAFER trial.[13] Several commercially available devices are directly marketed to consumers for detection of AF.[13] The results of SAFER will also guide clinicians on the appropriate course of action in AF identified through consumer-led screening.[13]

In addition to stroke prevention, there are other benefits to treating AF with anticoagulation, including improved survival and reduced risk of myocardial infarction.[11] Indeed, the STROKESTOP screening trial reported a marginally significant reduction in a revised composite primary end-point of stroke, systemic embolism, bleeding leading to hospitalisation and all cause death.[9] Another potential benefit of screening for AF is to reduce risk of cognitive decline and vascular dementia.[14-17]

For peer review only

In terms of harm, the major concern is risk of bleeding as a result of anticoagulation of people identified as being in AF. There is clear evidence in the trials of treatment of AF with anticoagulation that benefit outweighs harm,[11] but the ratio of benefit to harm of treatment might be different for people with AF identified through screening. For example, in the LOOP trial, the 20% relative risk reduction in stroke was largely offset by the 26% relative increase in risk of major bleeding.[8] This concern is reinforced by the results of recent trials of anticoagulation in sub-clinical atrial fibrillation and atrial high rate episodes (AHRES) detected as a result of implanted devices such as pacemakers, defibrillators and loop recorders (i.e not identified as a result of screening).[18 19] In the NOAH-AFNET6 trial, a non-significant 19% reduction in the primary efficacy outcome (composite of cardiovascular death, stroke and systemic embolism) was offset by a significant 31% increase in the risk of a safety outcome occurring (death from any cause or major bleeding).[18] In the ARTESIA trial, a significant 37% reduction in risk of stroke or systemic embolism was offset by a significant 36% increase in the risk of major bleeding.[19]

The aim of the SAFER trial is to determine if population screening for AF using a hand-held single-lead ECG device intermittently over a period of three weeks is effective and cost-effective at reducing stroke compared to usual care and to quantify other potential benefits and harms of screening. The design of the SAFER pilot trial (now successfully completed) has already been reported.[1] This protocol paper therefore focuses on changes in methods between the pilot and the main trial. The SPIRIT checklist provides the structure for this paper.[20]

METHODS AND ANALYSIS

Design

SAFER is a multi-centre randomised controlled trial. Randomisation is at the individual level with clustering by household (i.e., if there is more than one participant from the same address, they will be allocated to the same arm). This is a change from the original intention to randomise at the level of the general practice.[1] This decision was made during the internal pilot trial, when it became clear that remote delivery of the screening intervention greatly reduced the risk of contamination, so negating the value of cluster randomisation by practice. However, it was recognised that there would be a residual risk of contamination if members of the same household were in different arms of the trial. The first participant was randomised in March 2022. It is currently estimated that

For peer review only

randomisation will finish in April 2024 and follow-up will finish in March 2027. The trial design is summarised in Figure 1.

<<Figure 1. SAFER trial schema>>

Participants

Participant eligibility is unchanged from the pilot study, being people aged 70 years or older who are registered with participating general practices.[1] Those who are on the practice palliative care register or in a nursing or residential home are excluded, as are those already on anticoagulation therapy. Non-UK residents are excluded, as are people who have already consented to another trial that may affect participation in SAFER. People with a prior record of AF but not currently on anticoagulation are eligible as this may encourage anticoagulation use in these participants as was observed in STROKESTOP.[1] General practices are being recruited from throughout England. It is anticipated that about 195 practices will be involved.

Recruitment

Unlike in the pilot cluster randomised trial, where there was little gain in power from increasing sample size in each cluster, all eligible patients (as opposed to a random sample) are sent an invitation pack by their practice. This includes a consent form (see supplementary file 1) to be returned to the trial team either by post or online.

Randomisation

Randomisation is performed on-line at the Oxford Primary Care Clinical Trials Unit following return of consent forms, stratified by practice. Random permuted blocks ensure allocations are balanced at a ratio of 2:1 (control : intervention) in batches per practice. If there is more than one participant in the same household, they are randomised as a cluster to the same arm. In recognition that trial capacity would be limited primarily by how many participants could be screened, a 2:1 randomisation ratio was used to increase trial power for a given number of participants randomised to screening.

For peer review only

Baseline data

This is unchanged from the pilot trial, includes demographics and comorbidities, and is collected from the GP electronic medical records.[1]

Screening Intervention

This is unchanged from the pilot trial.[1] In brief, participants randomised to screening will receive a further postal invitation to participate in screening. Those who accept this invitation receive a call from the trial team to arrange delivery of the single-lead ECG device (Zenicor One, Zenicor medical systems AB) and instructions (written with online video available) and an offer of subsequent support by telephone on how to use it. They are asked to carry out screening four times a day for three weeks, and take additional traces if symptomatic (e.g. palpitations, dizziness). Each trace runs for 30 seconds. Participants transmit their recordings to a remote database using the mobile capability within the device. Each ECG is tagged with a unique participant ID number.

A proprietary algorithm (Cardiolund) analyses the ECG traces,[21] and those that show possible AF are reviewed by a cardiologist or cardiac technician. A second cardiologist performs additional review if there is uncertainty. AF is diagnosed if the rhythm is present continuously for 30 seconds. The screening results are returned to the practice, which notifies all participants of their results, and actively follows up those with AF or other significant diagnoses (e.g. ventricular tachycardia, high-degree atrioventricular block). Participating GPs receive initial training when the practice is set up for the trial. This includes a reminder that confirmation of the diagnosis of AF with a 12 lead ECG is not required for diagnosis of paroxysmal AF.[22] -They are offered further on line training on the National Institute for Health and Care Excellence (NICE) AF guidelines.[22] GPs are asked to provide a reason if they do not initiate anticoagulation for a participant diagnosed through screening.

Usual care

Participants assigned to the control arm will receive usual care, which might involve single time point opportunistic screening.

Follow up

For peer review only

The target follow up duration has been reduced from an average of five years (as per the pilot protocol)[1] to an average of four years per participant. This is to compensate for the delays imposed on the trial by COVID-19, and to lower the risk of control group contamination with risking direct marketing of AF detection devices directly to the public.[13] The revised sample size calculation (see below) takes this reduced length of follow up into account. The programme steering committee will review stroke rate in the whole trial population (i.e., not by treatment arm), and may recommend modifying follow up duration if stroke rates differ from what is expected (approximately 1% per annum).[9] Follow up will be by electronic health records (including GP records), Hospital Episode Statistics (HES), Office for National Statistics (ONS) mortality data and national disease registries accessed via NHS England and ORCHID database.[23] Participants are linked to these databases via a unique number (their NHS number). HES provides principal and secondary diagnosis codes for all hospital admissions in England. ONS mortality data includes date of death, and underlying and contributory causes of death for all deaths. National disease registries provide an alternative source for stroke and myocardial infarction to HES. A comparison of these sources suggests that data capture is more complete with combination of sources .[24]

Funding for longer term follow up will be sought. In particular, if AF screening is associated with reduction in dementia, the screening benefit will manifest over a longer time period.

Outcomes

The primary outcome is stroke. This includes stroke of any severity, but excludes events only labelled as transient ischaemic attack. For the primary endpoint, ischaemic and haemorrhagic stroke events will be combined.

Secondary outcomes include: all-cause death; cardiovascular death; major adverse cardiovascular event (composite of myocardial infarction, stroke and other hospital admissions for cardiovascular disease, including heart failure); myocardial infarction; ischaemic stroke; haemorrhagic stroke; major bleeding episode (defined as requiring hospital admission); new diagnosis of dementia; new diagnosis of depression. AF detection rates and anticoagulation uptake will be reported (principal outcomes of the internal pilot trial).

Outcome ascertainment will be restricted to data available from electronic health records without event adjudication. A comparison of routine versus adjudicated follow up in a vascular events

For peer review only

outcome trial found that specificity of routine data was high (over 99%), and that sensitivity was over 80% if transient ischaemic attack was excluded.[25] Furthermore, there was no difference in effect size between the two sources of data.[25] The sample size calculation below takes into account the 80% sensitivity, in that it is based on observed stroke rates in a trial where the follow up also relied on routinely available data.[9]

Sample size

The sample size calculation has been updated to reflect the changes in trial design, the result of a recent trial of screening for AF using the Zenicor One device,[9] the interim results of the internal pilot trial, and initial baseline findings from the main trial. In the STROKESTOP trial, an 8% reduction in risk of stroke was observed.[9] Due to higher uptake of screening in the intervention arm of SAFER, and the greater observed differences in AF detection rates between intervention and control as compared to STROKESTOP, a 12% relative risk reduction in stroke is now anticipated in SAFER. Assuming a household cluster size of 1.21 (from observed cluster size to date), a household intraclass correlation coefficient of 0.2,[26] and a 1% annual risk of stroke in the control arm,[9] this equates to needing 82,000 participants to detect a 12% relative reduction in risk of stroke after four years with 90% power. Overall, the target number of participants was reduced from 126,000 to 82,000, primarily as a result of the change from being a cluster randomised trial at the level of the practice to randomisation by household. Our experience in our feasibility and pilot studies (which will be reported separately) suggest that this number will be achievable.

Analysis

The intention-to-treat principle will guide data analysis (outcome in all eligible randomised participants will be compared between intervention and control). All eligible randomised participants will be included in the analysis, regardless of participation in screening.

The primary analysis will be conducted separately for the cluster randomised pilot trial and the main trial, with results then combined by fixed effect meta-analysis. Time-to-event modelling (i.e. a Cox proportional hazards model) will be used to obtain an estimate (hazard ratio) of the effect of screening on stroke risk (fatal and non-fatal), censoring other causes of death. Analysis time will be from date of randomisation.

For peer review only

Clustering (by practice for pilot trial participants and by household for main trial participants) will be accounted for using a robust sandwich estimator of the covariance matrix. The estimate of intervention effect will be adjusted for pre-specified baseline co-variates such as age and sex. Secondary outcomes will be analysed in a similar way.

For all analyses, we will test model assumptions. Should these be violated, flexible parametric survival models will be considered to model the change in hazard ratio over time.

A full statistical analysis plan will be lodged with the ISRCTN registration prior to data lock.

Economic analysis

To determine whether screening is cost-effective from the perspective of the NHS, we will adapt an existing economic model.[27] This will incorporate data from the SAFER trial, including outcomes such as mortality and cardiovascular endpoints, to determine incremental cost per QALY gained comparing screening versus no screening over a 4 year time horizon. The model parameters that do not come from the trial will be derived from updated literature reviews. We will extend the model to a life-time horizon, and consider the impact on cost-effectiveness of repeated screening at different time intervals and in different age groups.

Management and oversight

Management and oversight is delivered through the same structure as in the pilot trial.[1] The University of Cambridge and NHS Cambridgeshire & Peterborough Integrated Care Board (ICB) are co-sponsors. The trial management group meets monthly to review operational issues. The programme steering committee (PSC), which has an independent chair and four independent members, provides independent over-sight of the programme and acts as the Trial Steering Committee. An active risk register has been compiled in consultation with the funder and sponsors and will be monitored and updated throughout.

Patient and public involvement (PPI)

For peer review only

The same approach is being used as in the pilot trial.[1] In brief, we have engagement by PPI members as an investigator (Trudie Lobban, chief executive of the Atrial Fibrillation Association, (AFA)), and as contributors independent of the AFA.

ETHICS AND DISSEMINATION

Ethical approval has been provided by the London-Central NHS Research Ethics Committee (19/LO/1597).

In addition to peer-reviewed publications and presentation at conferences, public-friendly trial summary documents will be made available to participants at the end of the trial. Accessible reports will be generated for the UK National Screening Committee, commissioners and other decision makers. The pilot study protocol provides further details.[1]

Requests for pseudonymised data will be directed to the trial co-ordinator (Andrew Dymond using SAFER@medschl.cam.ac.uk) and will be considered by the investigators, in accordance with participant consent.

AUTHOR CONTRIBUTIONS

JM is the guarantor and drafted the manuscript with help from RNM. KW and AD are coordinating, planning, gaining ethical approval, conducting, and helping design the trial. JM, JB, NA, DE, RJ, JL, TL, ML, GL, BF, SG, SS, FRH and RJM undertook design, planning and are overseeing conduct of the trial. SH and AP as qualitative researchers contributed to the design of the intervention. MC helped design the trial. TL is a PPI representative who has informed the design, outcomes and dissemination plan. SM and HT designed the economic evaluation and will oversee its conduct. SK designed the statistical analysis and will oversee its conduct. MS contributed to the initial work on the trial design and led statistical methods. SK contributed to revision of the trial design and will lead on development of the statistical analysis plan and oversee progress. GL, PC, MM, WD and RP conducted and refined the cardiology review process of the intervention. The SAFER author group contributed to planning and design of the trial, applying for funding, writing of the protocol for the ethical approval and have oversight of the conduct of the trial. All authors reviewed and had the option to edit the final manuscript.

For peer review only

FUNDING STATEMENT

This SAFER trial is funded by the National Institute for Health and Care Research (NIHR) [Programme Grants for Applied Research Programme (Reference Number RP-PG-0217-20007)]. The views expressed are those of the author(s) and not necessarily those of the NIHR or the Department of Health and Social Care. SAFER is a contributor to / partner of AFFECT-EU receiving funding from the European Union's Horizon 2020 research and innovation Programme under grant agreement NO. 847770. RM and JL are supported by the Wellcome Trust as part of the Wellcome Trust PhD Programme for Primary Care Clinicians [grant number 203921/Z/16/Z]. AP is based in The Healthcare Improvement Studies Institute (THIS Institute), University of Cambridge. THIS Institute is supported by the Health Foundation, an independent charity committed to bringing about better health and healthcare for people in the UK. FDRH acknowledges support from NIHR ARC OTV and Oxford BRC (OUT). RJM is an NIHR Senior Investigator and acknowledges support from NIHR ARC OTV. NA is supported by the National Institute for Health and Care Research (NIHR) Greater Manchester Patient Safety Research Collaboration (GM PSRC) by the NIHR Applied Research Collaboration East Midlands (ARC EM). RJ is an NIHR-funded Academic Clinical Lecturer. The University of Cambridge has received salary support in respect of SJG from the NHS in the East of England through the Clinical Academic Reserve. BF received funding from the Medical Research Future Fund International Clinical Trial Collaboration Grant to perform SAFER-AUS as part of SAFER, and a NSW Health Senior Researcher Cardiovascular Grant for work in AF. GYHL is a National Institute for Health and Care Research (NIHR) Senior Investigator and co-principal investigator of the AFFIRMO project on multimorbidity in AF, which has received funding from the European Union's Horizon 2020 research and innovation programme under grant agreement No 899871. All the funders and sponsors had no involvement in the development of this protocol and will have no involvement in any aspect of the trialy itself. The views expressed are those of the author(s) and not necessarily those of the NHS, the Wellcome Trust, the NIHR or the UK Department of Health and Social Care.

COMPETING INTEREST STATEMENT

JM has performed consultancy work for BMS/Pfizer and Omron. FDRH reports occasional consultancy for BMS/Pfizer, Bayer and BI over the past 5 years. NA is a member of the UK National Screening Committee. MRC and MS are employed by Astrazeneca PLC, but at the time of involvement with the trial were employed by Universities (Kings College London and University of Leicester respectively), for which they still hold honorary contracts   RJM's employer the University

For peer review only

of Oxford receives consultancy and licencing payments from Omron and Sensyne for BP telemonitoring interventions. GYHL is a consultant and speaker for BMS/Pfizer, Boehringer Ingelheim, Daiichi-Sankyo, Anthos. No fees are received personally. SJG has received honoraria from Astra Zeneca for lectures at postgraduate educational meetings for primary care teams about type 2 diabetes. BF has received speaker fees, honoraria, and non-financial support from the BMS and Pfizer Alliance; grants to the Institution for investigator-initiated studies from the BMS and Pfizer Alliance; and loan devices for investigator-initiated studies from Alivecor: all were unrelated to the present trial but related to screening for AF.

ACKNOWLEDGEMENTS

We would like to acknowledge the support of:
Independent Programme Steering Committee: Professor Christian Mallen, University of Keele (chair); Professor Anthony Rudd, Kings College London (independent member); Professor Ann Marie Swart, University of East Anglia (independent member); Professor Andy Vail, University of Manchester (independent member); Dr Bob Ward (independent lay member)
Patient and public involvement representatives: Margaret Corbett; Jennifer Crockford; Trudie Lobban MBE (Founder & CEO of Atrial Fibrillation Association); Sheilah Rengert; Dr Bob Ward

REFERENCES

1. Williams K, Modi RN, Dymond A, et al. Cluster randomised controlled trial of screening for atrial fibrillation in people aged 70 years and over to reduce stroke: protocol for the pilot study for the SAFER trial. *BMJ Open* 2022;12(9):e065066. doi: 10.1136/bmjopen-2022-065066
2. Curry SJ, Krist AH, Owens DK, et al. Screening for Atrial Fibrillation With Electrocardiography: US Preventive Services Task Force Recommendation Statement. *Jama* 2018;320(5):478-84. doi: 10.1001/jama.2018.10321 [published Online First: 2018/08/09]
3. Kaasenbrood F, Hollander M, de Bruijn SH, et al. Opportunistic screening versus usual care for diagnosing atrial fibrillation in general practice: a cluster randomised controlled trial. *Br J Gen Pract* 2020;70(695):e427-e33. doi: 10.3399/bjgp20X708161 [published Online First: 2020/01/29]
4. Uittenbogaart SB, Verbiest-van Gurp N, Lucassen WAM, et al. Opportunistic screening versus usual care for detection of atrial fibrillation in primary care: cluster randomised controlled trial. *BMJ* 2020;370:m3208. doi: 10.1136/bmj.m3208

For peer review only

5. Lubitz SA, Atlas SJ, Ashburner JM, et al. Screening for Atrial Fibrillation in Older Adults at Primary Care Visits: VITAL-AF Randomized Controlled Trial. *Circulation* 2022;145(13):946-54. doi: doi:10.1161/CIRCULATIONAHA.121.057014

6. Fitzmaurice DA, Hobbs FD, Jowett S, et al. Screening versus routine practice in detection of atrial fibrillation in patients aged 65 or over: cluster randomised controlled trial. *Bmj* 2007;335(7616):383. doi: 10.1136/bmj.39280.660567.55 [published Online First: 2007/08/04]

7. Gladstone DJ, Wachter R, Schmalstieg-Bahr K, et al. Screening for Atrial Fibrillation in the Older Population: A Randomized Clinical Trial. *JAMA Cardiology* 2021 doi: 10.1001/jamacardio.2021.0038

8. Svendsen JH, Diederichsen SZ, Højberg S, et al. Implantable loop recorder detection of atrial fibrillation to prevent stroke (The LOOP Study): a randomised controlled trial. *The Lancet* 2021 doi: https://doi.org/10.1016/S0140-6736(21)01698-6

9. Svennberg E, Friberg L, Frykman V, et al. Clinical outcomes in systematic screening for atrial fibrillation (STROKESTOP): a multicentre, parallel group, unmasked, randomised controlled trial. *The Lancet* 2021 doi: https://doi.org/10.1016/S0140-6736(21)01637-8

10. Ganesan AN, Chew DP, Hartshorne T, et al. The impact of atrial fibrillation type on the risk of thromboembolism, mortality, and bleeding: a systematic review and meta-analysis. *Eur Heart J* 2016;37(20):1591-602. doi: 10.1093/eurheartj/ehw007 [published Online First: 2016/02/19]

11. Aguilar MI, Hart R. Oral anticoagulants for preventing stroke in patients with non-valvular atrial fibrillation and no previous history of stroke or transient ischemic attacks. *Cochrane Database of Systematic Reviews* 2005(3) doi: 10.1002/14651858.CD001927.pub2

12. Go AS, Reynolds K, Yang J, et al. Association of Burden of Atrial Fibrillation With Risk of Ischemic Stroke in Adults With Paroxysmal Atrial Fibrillation: The KP-RHYTHM Study. *JAMA Cardiology* 2018;3(7):601-08. doi: 10.1001/jamacardio.2018.1176

13. Brandes A, Stavrakis S, Freedman B, et al. Consumer-Led Screening for Atrial Fibrillation: Frontier Review of the AF-SCREEN International Collaboration. *Circulation* 2022;146(19):1461-74. doi: 10.1161/circulationaha.121.058911 [published Online First: 2022/11/08]

14. Singh-Manoux A, Fayosse A, Sabia S, et al. Atrial fibrillation as a risk factor for cognitive decline and dementia. *European Heart Journal* 2017;38(34):2612-18.

15. Bezabhe WM, Bereznicki LR, Radford J, et al. Oral Anticoagulant Treatment and the Risk of Dementia in Patients With Atrial Fibrillation: A Population-Based Cohort Study. *Journal of the American Heart Association* 2022;11(7):e023098. doi: doi:10.1161/JAHA.121.023098

For peer review only

16. Friberg L, Andersson T, Rosenqvist M. Less dementia and stroke in low-risk patients with atrial fibrillation taking oral anticoagulation. *European Heart Journal* 2019;40(28):2327-35. doi: 10.1093/eurheartj/ehz304

17. Mavaddat N, Roalfe A, Fletcher K, et al. Warfarin versus aspirin for prevention of cognitive decline in atrial fibrillation: randomized controlled trial (Birmingham Atrial Fibrillation Treatment of the Aged Study). *Stroke* 2014;45(5):1381-86.

18. Kirchhof P, Toennis T, Goette A, et al. Anticoagulation with Edoxaban in Patients with Atrial High-Rate Episodes. *New England Journal of Medicine* 2023;389(13):1167-79. doi: 10.1056/NEJMoa2303062

19. Healey JS, Lopes RD, Granger CB, et al. Apixaban for Stroke Prevention in Subclinical Atrial Fibrillation. *New England Journal of Medicine* 2023;390(2):107-17. doi: 10.1056/NEJMoa2310234

20. Chan AW, Tetzlaff JM, Gøtzsche PC, et al. SPIRIT 2013 explanation and elaboration: guidance for protocols of clinical trials. *Bmj* 2013;346:e7586. doi: 10.1136/bmj.e7586 [published Online First: 2013/01/11]

21. Cardiolund. Cardiac Analysis 2023 [Available from: https://www.cardiolund.com/ accessed 31/07/2023.

22. National Institute for Health and Care Excellence. Atrial fibrillation: diagnosis and management (ng196): National Institute of Health and Care Excellence, 2021.

23. Nuffield Department of Primary Care Health Sciences. ORCHID: University of Oxford; 2023 [Available from: https://orchid.phc.ox.ac.uk/ accessed 13/11/2023.

24. Herrett E, Shah AD, Boggon R, et al. Completeness and diagnostic validity of recording acute myocardial infarction events in primary care, hospital care, disease registry, and national mortality records: cohort study. *BMJ : British Medical Journal* 2013;346:f2350. doi: 10.1136/bmj.f2350

25. Harper C, Mafham M, Herrington W, et al. Comparison of the Accuracy and Completeness of Records of Serious Vascular Events in Routinely Collected Data vs Clinical Trial–Adjudicated Direct Follow-up Data in the UK: Secondary Analysis of the ASCEND Randomized Clinical Trial. *JAMA Network Open* 2021;4(12):e2139748-e48. doi: 10.1001/jamanetworkopen.2021.39748

26. Ukoumunne OC, Gulliford MC, Chinn S, et al. Methods for evaluating area-wide and organisation-based interventions in health and health care: a systematic review. *Health technology assessment (Winchester, England)* 1999;3(5):iii-92. [published Online First: 2000/09/12]

For peer review only

27. Welton NJ, McAleenan A, Thom HH, et al. Screening strategies for atrial fibrillation: a systematic review and cost-effectiveness analysis. *Health Technology Assessment* 2017;21(29)

For peer review only

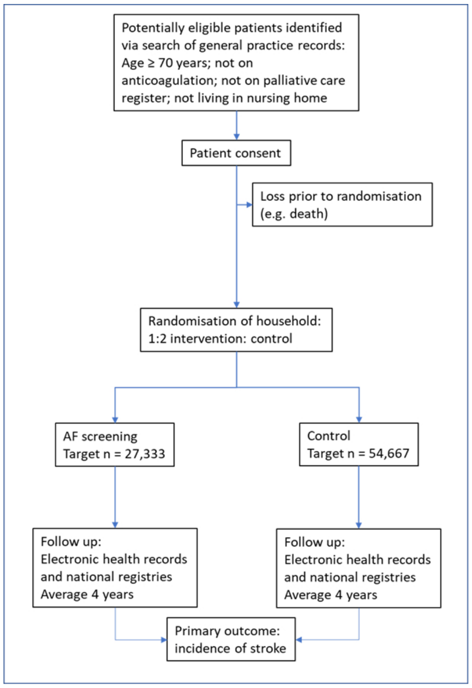

Figure 1. SAFER trial schema

225x180mm (144 x 144 DPI)

UNIVERSITY OF CAMBRIDGE
Public Health and Primary Care
The Primary Care Unit

SAFER trial

SAFER Trial
Consent Form

Version 2.0 16-12-2021

Please complete and return this form only if you wish to join the SAFER Trial

Title: The SAFER Trial – Screening for Atrial Fibrillation with ECG to Reduce stroke

Chief Investigator: Professor Jonathan Mant, University of Cambridge

Ethics Reference number: 19/LO/1597 **IRAS project ID:** 272184

If you are willing to take part in the SAFER Trial, please read the following statements and if you agree, sign and date overleaf.

1	I have read and understood the Participant Information Sheet version 2.0, dated 16/12/2021 (remote) for the above trial. I have had the opportunity to ask questions and I am satisfied with the answers and explanations provided.
2	I understand that my participation in this trial is voluntary and that I am free to withdraw at any time, without giving a reason and without my medical care or legal rights being affected.
3	I understand that information from my medical records will be available to the research team as part of the trial.
4	I consent to my trial data being linked to Hospital Episodes Statistics (HES), civil registration mortality data, Sentinel Stroke National Audit Programme (SSNAP) and Myocardial Ischaemia National Audit Project (MINAP). This may involve sharing my personal data with these bodies. I understand that information held and managed by NHS Digital and the registries may be used in order to provide information about my health status (including after my death), my GP practice and my address (should I move). I understand that these details will be used for research purposes only. It is possible that in the future the research team may need to link to another health record or registry not listed that they consider to be relevant to the purposes of the research and I agree to this.
5	I understand that sections of my medical notes or information related directly to my participation in this trial may be looked at by responsible individuals from the sponsors, regulatory authorities and research personnel where it is relevant to my taking part in this research. I give permission for these individuals to have access to my records.
6	I understand that my GP will be informed of my participation in this trial.
7	I understand that my unidentifiable trial data will be shared with other researchers, both internal and external to this trial, and with commercial partners. These parties may be outside the European Economic Area.
8	I understand that I may be contacted about future, related research studies, and that I am under no obligation to take part.
9	I agree to participate in this trial.

example - do not use
For peer review only

For peer review only - http://bmjopen.bmj.com/site/about/guidelines.xhtml

UNIVERSITY OF CAMBRIDGE
Public Health and Primary Care
The Primary Care Unit

SAFER trial

By signing this form you are consenting that you agree with all of the statements listed, and that the details listed below are correct.

Name of participant	Signature	Date

Please check the details below and amend/complete accordingly, then return this form to the trial team using the Freepost envelope enclosed. Alternatively you can complete this consent form online – please see the covering letter enclosed for instructions.

As the trial will be conducted remotely, it will be helpful if you could please supply phone number(s) and an email address. By providing these details you are agreeing to be contacted by the trial team via these methods (email, phone call, SMS text message) for the purposes of the trial.

Title:	
First name:	
Surname:	
Date of birth (dd/mm/yyyy):	
Gender (M/F/Mx):	
Address:	
Postcode:	
**Home Tel.:**	
**Mobile no.:**	
**Email:**	
NHS no:	
GP Practice name:<br>**Please note:** if this is **not** your current practice and you have recently moved practice, you will not be able to take part at this point. It is possible that your new practice may take part in the future.	

The trial team will return a copy of this consent form to your GP practice for their records. If you would like a copy of your completed consent form please contact the trial team.

The trial team will only use these details in order to contact you for the purposes stated.

1x copy to be retained by the research team; 1x copy to be sent to the participant's GP practice.

example - do not use
For peer review only

For peer review only - http://bmjopen.bmj.com/site/about/guidelines.xhtml

Reporting checklist for protocol of a clinical trial.

Based on the SPIRIT guidelines.

Administrative information	Reporting Item	Page Number
Title	Descriptive title identifying the study design, population, interventions, and, if applicable, trial acronym	1
Trial registration	Trial identifier and registry name. If not yet registered, name of intended registry	4
Trial registration: data set	All items from the World Health Organization Trial Registration Data Set	4
Protocol version	Date and version identifier	n/a
Funding	Sources and types of financial, material, and other support	12-13
Roles and responsibilities: contributorship	Names, affiliations, and roles of protocol contributors	1-3, 12

For peer review only

Roles and responsibilities: sponsor contact information	Name and contact information for the trial sponsor	11
Roles and responsibilities: sponsor and funder	Role of study sponsor and funders, if any, in study design; collection, management, analysis, and interpretation of data; writing of the report; and the decision to submit the report for publication, including whether they will have ultimate authority over any of these activities	11-12
Roles and responsibilities: committees	Composition, roles, and responsibilities of the coordinating centre, steering committee, endpoint adjudication committee, data management team, and other individuals or groups overseeing the trial, if applicable (see Item 21a for data monitoring committee)	11, 13
**Introduction**		
Background and rationale	Description of research question and justification for undertaking the trial, including summary of relevant studies (published and unpublished) examining benefits and harms for each intervention	6-7
Background and rationale: choice of comparators	Explanation for choice of comparators	6-9

For peer review only

Objectives	Specific objectives or hypotheses	7
Trial design	Description of trial design including type of trial (eg, parallel group, crossover, factorial, single group), allocation ratio, and framework (eg, superiority, equivalence, non-inferiority, exploratory)	7
**Methods: Participants, interventions, and outcomes**		
Study setting	Description of study settings (eg, community clinic, academic hospital) and list of countries where data will be collected. Reference to where list of study sites can be obtained	7-8, 12
Eligibility criteria	Inclusion and exclusion criteria for participants. If applicable, eligibility criteria for study centres and individuals who will perform the interventions (eg, surgeons, psychotherapists)	7-8
Interventions: description	Interventions for each group with sufficient detail to allow replication, including how and when they will be administered	8-9
Interventions: modifications	Criteria for discontinuing or modifying allocated interventions for a given trial participant (eg, drug dose change in response to harms, participant request, or improving / worsening disease)	8-9

For peer review only

Interventions: adherance	Strategies to improve adherence to intervention protocols, and any procedures for monitoring adherence (eg, drug tablet return; laboratory tests)	9
Interventions: concomitant care	Relevant concomitant care and interventions that are permitted or prohibited during the trial	8-9
Outcomes	Primary, secondary, and other outcomes, including the specific measurement variable (eg, systolic blood pressure), analysis metric (eg, change from baseline, final value, time to event), method of aggregation (eg, median, proportion), and time point for each outcome. Explanation of the clinical relevance of chosen efficacy and harm outcomes is strongly recommended	9
Participant timeline	Time schedule of enrolment, interventions (including any run-ins and washouts), assessments, and visits for participants. A schematic diagram is highly recommended (see Figure)	8-9
Sample size	Estimated number of participants needed to achieve study objectives and how it was determined, including clinical and statistical assumptions supporting any sample size calculations	9-10

For peer review only

Recruitment	Strategies for achieving adequate participant enrolment to reach target sample size	7-8
**Methods: Assignment of interventions (for controlled trials)**		
Allocation: sequence generation	Method of generating the allocation sequence (eg, computer-generated random numbers), and list of any factors for stratification. To reduce predictability of a random sequence, details of any planned restriction (eg, blocking) should be provided in a separate document that is unavailable to those who enrol participants or assign interventions	8
Allocation concealment mechanism	Mechanism of implementing the allocation sequence (eg, central telephone; sequentially numbered, opaque, sealed envelopes), describing any steps to conceal the sequence until interventions are assigned	n/a
Allocation: implementation	Who will generate the allocation sequence, who will enrol participants, and who will assign participants to interventions	8
Blinding (masking)	Who will be blinded after assignment to interventions (eg, trial participants, care providers, outcome assessors, data analysts), and how	n/a

For peer review only

Blinding (masking): emergency unblinding	If blinded, circumstances under which unblinding is permissible, and procedure for revealing a participant's allocated intervention during the trial	n/a
**Methods: Data collection, management, and analysis**		
Data collection plan	Plans for assessment and collection of outcome, baseline, and other trial data, including any related processes to promote data quality (eg, duplicate measurements, training of assessors) and a description of study instruments (eg, questionnaires, laboratory tests) along with their reliability and validity, if known. Reference to where data collection forms can be found, if not in the protocol	8-9
Data collection plan: retention	Plans to promote participant retention and complete follow-up, including list of any outcome data to be collected for participants who discontinue or deviate from intervention protocols	9
Data management	Plans for data entry, coding, security, and storage, including any related processes to promote data quality (eg, double data entry; range checks for data values). Reference to where details of data	8, 11

For peer review only

	management procedures can be found, if not in the protocol	
Statistics: outcomes	Statistical methods for analysing primary and secondary outcomes. Reference to where other details of the statistical analysis plan can be found, if not in the protocol	10-11
Statistics: additional analyses	Methods for any additional analyses (eg, subgroup and adjusted analyses)	10-11
Statistics: analysis population and missing data	Definition of analysis population relating to protocol non-adherence (eg, as randomised analysis), and any statistical methods to handle missing data (eg, multiple imputation)	10
**Methods: Monitoring**		
Data monitoring: formal committee	Composition of data monitoring committee (DMC); summary of its role and reporting structure; statement of whether it is independent from the sponsor and competing interests; and reference to where further details about its charter can be found, if not in the protocol. Alternatively, an explanation of why a DMC is not needed	11, 13
Data monitoring: interim analysis	Description of any interim analyses and stopping guidelines, including who will have access to these interim results and make the final decision to terminate the trial	11, 13

For peer review only

Harms	Plans for collecting, assessing, reporting, and managing solicited and spontaneously reported adverse events and other unintended effects of trial interventions or trial conduct	11
Auditing	Frequency and procedures for auditing trial conduct, if any, and whether the process will be independent from investigators and the sponsor	11
**Ethics and dissemination**		
Research ethics approval	Plans for seeking research ethics committee / institutional review board (REC / IRB) approval	11-12
Protocol amendments	Plans for communicating important protocol modifications (eg, changes to eligibility criteria, outcomes, analyses) to relevant parties (eg, investigators, REC / IRBs, trial participants, trial registries, journals, regulators)	11
Consent or assent	Who will obtain informed consent or assent from potential trial participants or authorised surrogates, and how (see Item 32)	8
Consent or assent: ancillary studies	Additional consent provisions for collection and use of participant data and biological specimens in ancillary studies, if applicable	n/a

For peer review only

Confidentiality	How personal information about potential and enrolled participants will be collected, shared, and maintained in order to protect confidentiality before, during, and after the trial	11-12
Declaration of interests	Financial and other competing interests for principal investigators for the overall trial and each study site	12-13
Data access	Statement of who will have access to the final trial dataset, and disclosure of contractual agreements that limit such access for investigators	12
Ancillary and post trial care	Provisions, if any, for ancillary and post-trial care, and for compensation to those who suffer harm from trial participation	n/a
Dissemination policy: trial results	Plans for investigators and sponsor to communicate trial results to participants, healthcare professionals, the public, and other relevant groups (eg, via publication, reporting in results databases, or other data sharing arrangements), including any publication restrictions	11
Dissemination policy: authorship	Authorship eligibility guidelines and any intended use of professional writers	12

For peer review only

Dissemination policy: reproducible research	Plans, if any, for granting public access to the full protocol, participant-level dataset, and statistical code	12

Appendices

Informed consent materials	Model consent form and other related documentation given to participants and authorised surrogates	Supplemental material
Biological specimens	Plans for collection, laboratory evaluation, and storage of biological specimens for genetic or molecular analysis in the current trial and for future use in ancillary studies, if applicable	n/a

For peer review only
